# Genetic Basis and Simulated Breeding Strategies for Enhancing Soybean Seed Protein Content Across Multiple Environments

**DOI:** 10.3390/plants14142117

**Published:** 2025-07-09

**Authors:** Xu Sun, Bo Hu, Wen-Xia Li, Hai-Long Ning

**Affiliations:** 1 Key Laboratory of Soybean Biology, Ministry of Education, Northeast Agricultural University, Harbin 150038, China; 15546109835@163.com (X.S.); hb554512341@163.com (B.H.); 2 Key Laboratory of Soybean Biology and Breeding/Genetics, Ministry of Agriculture, Northeast Agricultural University, Harbin 150038, China

**Keywords:** soybean, protein content, QTL, design breeding, candidate genes

## Abstract

Soybeans are a primary source of plant-based protein, with seeds containing approximately 40% protein—a key quality trait. Selecting superior hybrid combinations and managing progeny effectively are crucial for developing high-protein soybean varieties. Using a recombinant inbred line population (RIL3613) derived from Dongnong L13 × Heihe 36 and its previously constructed high-density genetic linkage map, QTLs and QTL × environment interactions (QEIs) associated with seed protein content (SPC) were identified through the bi-parental population (BIP) model and multi-environment trials (MET) model in QTL IciMapping v4.2. Candidate genes were then predicted via sequence alignment and haplotype analysis between the parents. Finally, simulated breeding was conducted using the B4L function in the In Silico Breeding (ISB) module of the Blib platform to determine optimal breeding strategies across diverse environments. The analysis identified 19 QTLs associated with SPC and 97 QEIs linked to SPC. These QTLs collectively explained 84.442% of the phenotypic variance, with four QTLs exhibiting significant contributions. A key candidate gene, *Glyma.12G231400*, associated with soybean SPC, was predicted within the 38,995,090–39,293,825 bp interval on chromosome 12. Across 11 environments, three to six optimal breeding schemes were selected, all employing modified pedigree selection. These findings enhance our understanding of the genetic basis of soybean protein formation and provide technological support for molecular breeding for seed quality improvement.

## 1. Introduction

Soybean is one of the world’s most important crops and a primary source of protein and oil for humans [1,2]. China has a long history of soybean production. With the rapid development of modern soybean cultivation in Northeast China, soybeans have become a hallmark of the region’s agricultural economic history [3]. The genetic improvement of soybean seed protein is crucial in terms of meeting the demands of the growing global population [4]. A primary objective in soybean breeding is to enhance protein content [5].

Soybean protein content is a complex quantitative trait influenced by environmental conditions and controlled by multiple genes [6]. Most studies indicate that while seed composition is primarily governed by genetic factors, it is also affected by abiotic and biotic factors [7]. The protein and oil content in seeds of the same variety can vary across years or under different environmental conditions within the same year [8]. Molecular geneticists and breeders commonly use populations derived from biparental crosses to select new varieties and map quantitative trait loci (QTL) for target traits [9]. Recent research has focused on identifying QTLs for protein content and mining genes using linkage analysis and genome-wide association studies (GWAS) with high-density genetic maps. These approaches have been instrumental in elucidating the genetic architecture of soybean seed protein and facilitating variety improvement [10]. According to the SoyBase database, 241 QTLs influencing soybean SPC had been identified by 2018, with additional QTLs discovered in subsequent years. Karikari et al. [11] constructed a genetic map using 2267 bin markers, identifying 25 protein QTLs. By integrating these findings with transcriptomic data, they pinpointed four candidate genes associated with protein synthesis. Zhong et al. [12] used a high-density genetic map to evaluate QTLs for protein content, detecting 44 major and stable QTLs. Cunicelli et al. [13] analyzed 138 recombinant inbred lines across six environments, identifying 21 QTLs for traits including yield, protein, oil content, methionine, and threonine, with four linked to protein. Seo et al. [14] identified 12 seed protein content-related QTLs. Further investigation of candidate genes within major-effect QTLs could provide deeper insights into the genetic basis of SPC. Lee et al. [15] identified 192 co-linear protein QTLs, forming six hotspot regions, and detected eight genes that are highly expressed during seed maturation. Yang et al. [16] mapped protein content to a 15 kb interval using 195 chromosome segment substitution lines and, in conjunction with transcriptomic data, designated *Glyma.15G049200* as a candidate gene. Salas et al. [17] mapped two stable protein QTLs, i.e., qPro-10-1 and qPro-14-1. Fliege et al. [9] performed fine mapping of the cq-Seed protein-003 QTL on chromosome 20, identifying *Glyma.20G85100* as a gene related to soybean seed protein content.

Large-scale genomic sequencing, high-density linkage analysis, genome-wide association studies, and extensive functional genomics research have made designed breeding a tangible reality. Breeders are gradually moving away from traditional methods and adopting the “priori design” concept [18]. This shift is driven by the long breeding cycle of soybeans. Currently, parental line selection still heavily relies on breeders’ experience and intuition. Designed breeding effectively addresses these challenges. It aims to control all allelic variations of genes which are essential for agronomic traits. This control becomes possible through precise genetic maps, high-resolution chromosome single-nucleotide analysis, and extensive phenotypic evaluations [19].

Designed breeding has proven effective in plant breeding. Wei et al. [20] combined marker-assisted selection with multiple resistance screening. After several rounds of hybridization, they aggregated six target genes and developed a promising restorer line: Guihui5501. This line exhibited heavy grain, good quality, and tolerance to both biotic and abiotic stresses. To develop high oleic acid soybean varieties, Nan et al. [21] analyzed the FAD2-1A and FAD2-1B haplotypes—key factors in increasing oleic acid content—in 1250 soybean materials and developed two molecular markers. Using marker-assisted selection, they identified line 435, which had an oleic acid content of 91.03%. Line 435 was then used as the donor parent, with the superior soybean variety Hainong 51 serving as the recurrent parent. After three backcrosses, a single plant with high oleic acid content (75%) and high yield was obtained. These case studies highlight how parental line selection and breeding strategies determine the success of breeding objectives [22]. In recent years, methods such as BLUP and genomic selection (GS) have been used to estimate parental breeding potential and guide selection in crop improvement [23]. Additionally, parental selection can be based on predicted performance. Zhong et al. [24] proposed selecting inbred line parents based on the projected performance of the best offspring from a cross, termed “superior progeny value.” When designing breeding schemes, breeders must choose the optimal strategy from multiple options before initiating actual breeding. Computer simulations effectively compare multiple breeding methods and identify the most efficient scheme for generating the target genotype, thereby saving time, land, and labor costs. These simulations incorporate assumptions about population and quantitative genetics, influencing the final breeding plan [25]. Additionally, they generate extensive data that may be difficult to obtain through empirical experiments or theoretical models, helping validate proposed theories or models [26]. By leveraging parental molecular data and genomic prediction models, simulations can create segregating populations from virtual crosses, enabling the prediction of the most promising populations before conducting actual field crosses [27]. Bančič et al. [28] recently developed AlphaSimR, a software package that allows breeders to design and simulate breeding schemes independently. Zhang et al. [29] recently developed Blib, a multi-module simulation platform capable of handling more complex genetic effects and models than existing tools, making it suitable for modeling, simulating, and predicting genetic breeding processes in diploid species. Building on the Blib platform, Wang et al. [30] proposed a wheat breeding design method that integrates known QTL information with computer simulations. Potential crosses within a GWAS panel can be evaluated based on the relative frequency of target genotypes, trait correlations in simulated progeny, and genetic gains in selected progeny. By optimizing parental selection, progeny population size, and selection schemes, both yield and grain quality can be improved simultaneously. Applying this design method enables the identification of the most promising crosses and selection strategies before field trials, enhancing the predictability and efficiency of breeding programs.

Based on previous studies, research on identifying QTLs and potential candidate genes associated with protein content is extensive. However, the effects of different haplotypes of candidate genes on soybean SPC remain underexplored. Additionally, the use of genetic information to develop models and assess the breeding potential of soybean SPC is limited.

In our previous studies, a recombinant inbred line (RIL) population, RIL3613, was constructed and used to map QTLs for SPC primarily based on an SSR linkage map [31,32,33]. However, due to a lack of fine mapping, these QTLs could not be applied in molecular breeding. To identify optimal breeding strategies for SPC in RIL3613, the present study conducted a linkage analysis using a high-density SNP linkage map to map SPC-related QTLs and QEI across the whole genome. Key candidate genes were identified through parental sequence comparison and haplotype analysis. A genetic model was constructed using the ISB plant breeding simulation platform, incorporating QEI data. Breeding simulations were then conducted with the RIL3613 population as parental lines to determine optimal breeding strategies for diverse environments.

This study aims to provide a theoretical foundation and technical support for the genetic improvement of soybeans.

## 2. Results

### 2.1. Phenotypic Variation Analysis

The phenotypic data of 120 lines from the RIL3613 population across 22 environments were analyzed. A descriptive analysis showed that the absolute values of kurtosis and skewness were <1 in all environments except E09, E10, E17, E19, E20, and E21, indicating a normal distribution of protein content (Table 1, Figure S1). The protein content in RIL3613 spanned the parental range, suggesting transgressive segregation. The coefficient of variation ranged from 2.04% to 5.34%. Analysis of variance revealed highly significant effects of environment, genotype, and genotype × environment interaction, demonstrating that protein content is influenced by both genetic and environmental factors (Table 2). The broad-sense heritability was high (82.3%), indicating that genetic effects primarily drive variation in soybean protein content.

### 2.2. QTL Mapping for Candidate Gene Prediction

**Figure 1:**
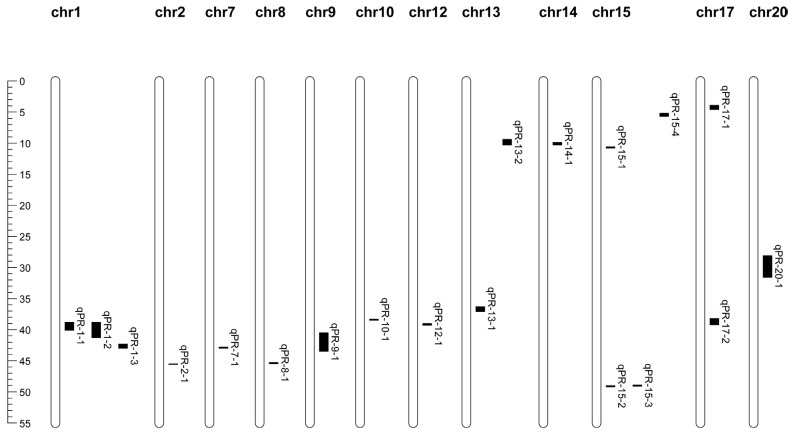
Distribution of QTLs for protein content identified in RIL3613 on genome map.

Using the bin map developed previously [34], the ICIM method in the BIP module identified 19 QTLs associated with protein content, distributed across 13 chromosomes, with each chromosome harboring one to four QTLs. These QTLs explained 0.35% to 15.83% of the phenotypic variance, collectively accounting for 84.442% of the total phenotypic variation (Figure 1, Table S1). Notably, four QTLs (qPR-1-1, qPR-1-3, qPR-12-1, and qPR-14-1) contributed over 10% to phenotypic variation and are considered major effective QTLs for soybean SPC (Table 3).

### 2.3. Candidate Gene Prediction

Potential candidate genes within these four QTL regions (qPR-1-1, qPR-1-3, qPR-12-1, and qPR-14-1) were identified, corresponding to genomic intervals of 38.76–41.33 Mb on Chr01, 42.34–43.01 Mb on Chr01, 39.00–39.29 Mb on Chr12, and 9.90–10.29 Mb on Chr14. A total of 147 genes were identified. Resequencing data from parental lines revealed 52 genes with non-synonymous mutations. Functional annotation of these 52 genes led to the identification of three candidate genes (see Table 4 and Table 5).

### 2.4. Haplotype Analysis and Validation

**Figure 2:**
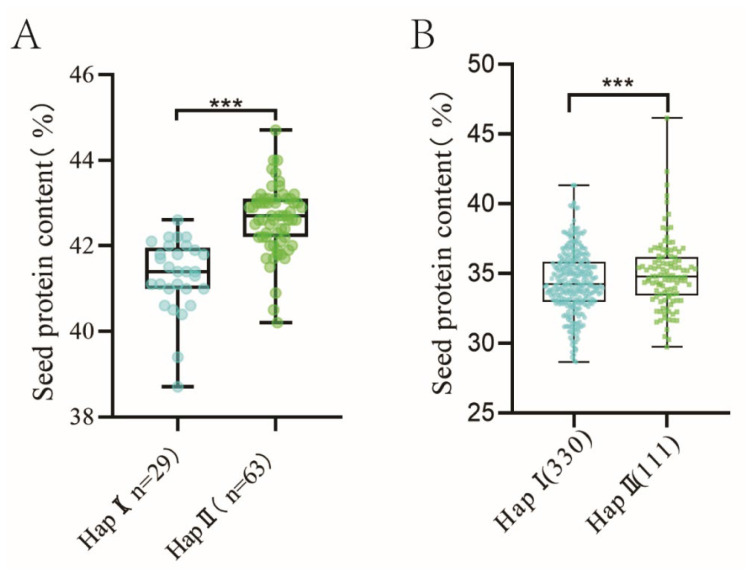
(**A**): Identification of *Glyma.12G231400* through haplotype analysis in the RIL3613 population (*** indicates *p* < 0.001). (**B**): Haplotype validation of *Glyma.12G231400* in germplasm resource populations. (*** indicates *p* < 0.001).

To further validate the function of the candidate genes, two haplotypes were defined for each of the three candidate genes based on missense mutations in the parental CDS. For a list of primers targeting these mutation sites, see Table S2. The corresponding fragments were then amplified via PCR and sequenced from the DNA of 92 individual lines in the RIL3613 population. Haplotype analysis of the candidate genes was performed using the sequencing results and SPC phenotypic data from the RIL3613 population. This analysis revealed a significant difference in the SPC trait between haplotypes only for the *Glyma.12G231400* gene (Figure 2A). This suggests that HapII of *Glyma.12G231400* may be a superior haplotype for enhancing soybean protein content. Consequently, *Glyma.12G231400* was designated as the final candidate gene.

To assess the regulatory effect of the candidate gene on protein content across diverse genetic backgrounds, a haplotype analysis of *Glyma.12G231400* was conducted using genotypic and phenotypic data from 2898 soybean germplasm resources in the SoyOmics database. The results showed that the HapⅠ haplotype was present in 1612 accessions, with SPC phenotypic data available for 330 accessions, whereas the HapII haplotype was found in 453 accessions, with SPC data available for 111 accessions. The SPC of HapII remained significantly higher than that of HapⅠ (Figure 2B), further confirming the regulatory role of *Glyma.12G231400* in soybean SPC.

### 2.5. Haplotype Analysis and Validation

**Figure 3:**
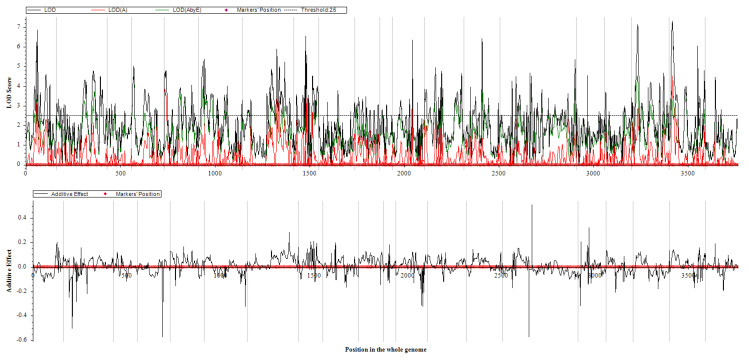
LOD and additive effect curves of QTL for protein content.

Using the BIP model in QTL IciMapping v4.2 [35], we identified 19 QTLs across 11 of the 22 analyzed environments (E08, E09, E11, E14, E15, E16, E17, E18, E19, E20, and E21). To apply QEI to simulation breeding, we employed the MET model to map QEIs for soybean SPC across these 11 environments in the RIL3613 population. A total of 97 QEIs associated with SPC, exhibiting additive-by-environment (A × E) effects, were detected across 20 chromosomes. LOD scores for these QEIs ranged from 2.5153 to 7.3106, with each QEI explaining 0.63% to 2.49% of the phenotypic variance (Figure 3 and Figure S2, Table S3). The phenotypic contribution of each QEI was relatively low, indicating that most were minor-effect QEIs. The phenotypic variance explained by environmental effects (PVE (A × E)) was also small, suggesting that these QEIs are relatively stable and suitable for breeding simulations.

### 2.6. Analysis of Simulated Breeding Results

Based on the additive effects of QEI across diverse environments, the synergistic allele among the two QEI alleles was designated as the superior allele (SA). The number of SA and genotypic values varied across environments for the same variety. Moreover, significant differences in SA numbers and genotypic values were observed among varieties within each environment (Table S4). Consequently, breeding simulations were conducted separately for each environment.

**Figure 4:**
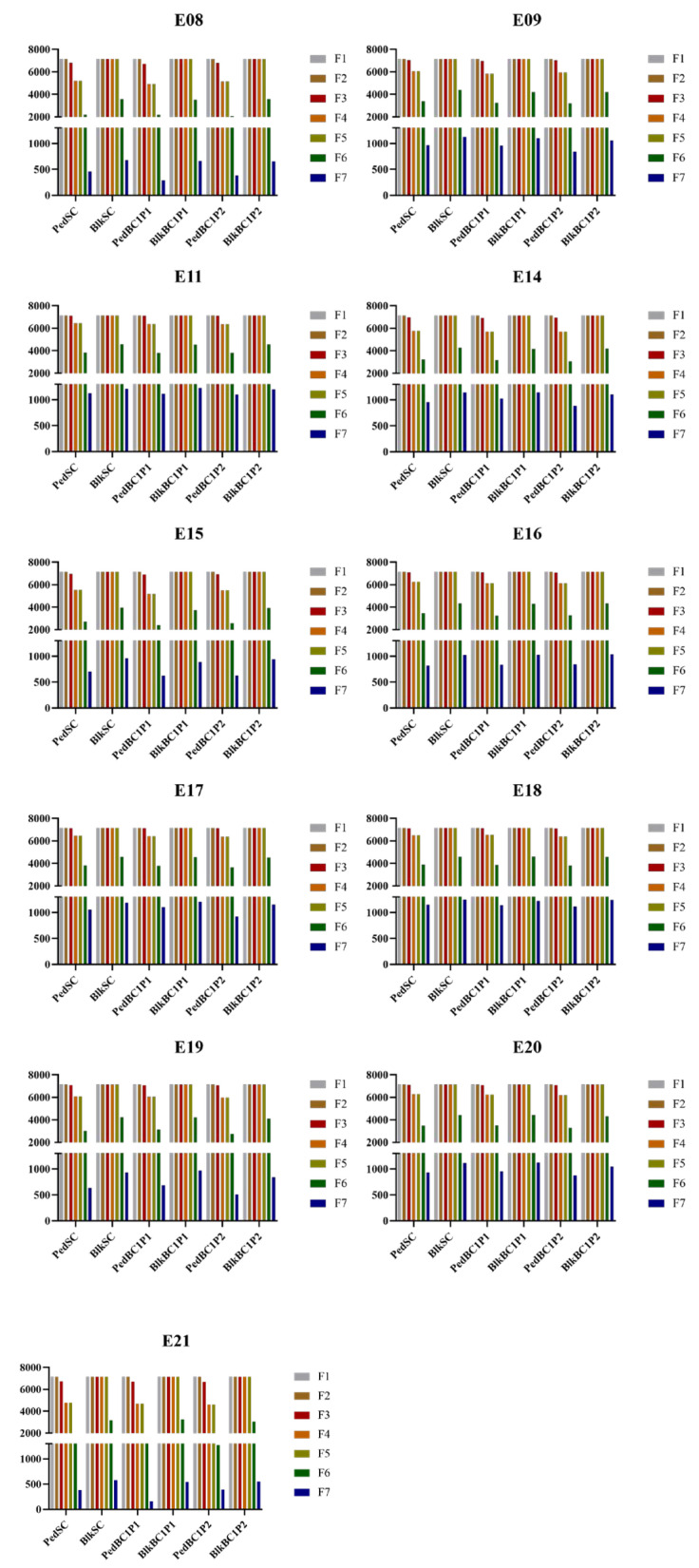
Selection of hybrid combinations across seven breeding generations. The x axis represents breeding schemes, the y axis denotes the number of hybrid combinations retained per breeding generation, and the bars represent each breeding generation. The six breeding schemes, from left to right, Pedigree Single Cross (PedSC), Bulk Single Cross (BlkSC), Pedigree Backcross 1 Parent 1 (PedBC1P1)/Pedigree Backcross 1 Parent 2 (PedBC1P2), and Bulk Backcross 1 Parent 1 (BlkBC1P1)/Bulk Backcross 1 Parent 2 (BlkBC1P2). E08 et al. represents the simulation under this environment.

**Figure 5:**
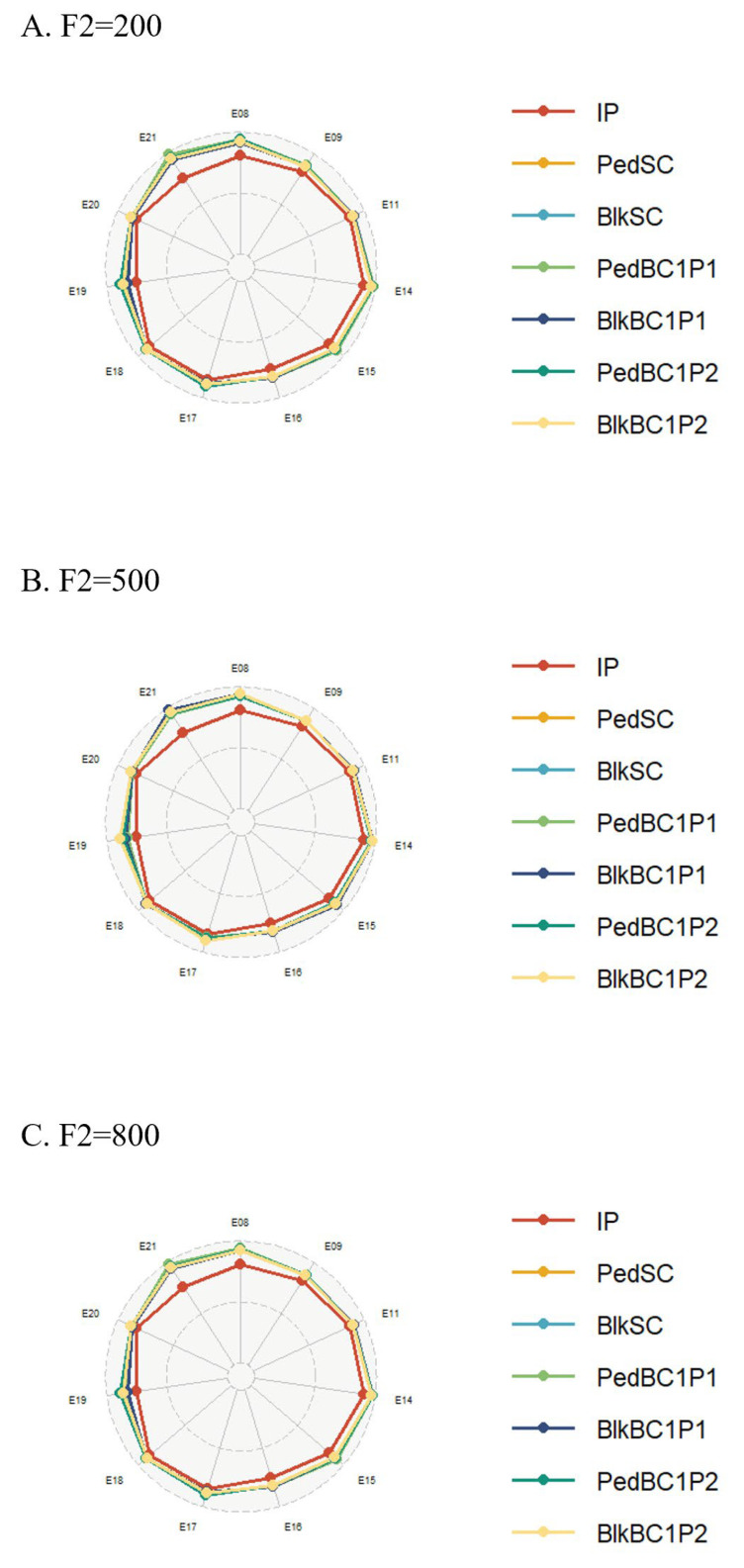
Comparison of the population mean of the progeny population with the initial population. (**A**–**C**) represents different F2 generation planting scale. IP represents the initial population. E08 et al. represents the simulation under this environment.

Using the acquired QEI data and genotypic information of individual lines in the RIL3613 population, the initial parental population and genetic models were constructed. Breeding schemes were simulated using the B4L function of ISB. The simulation results included the number of hybrid combinations retained after each selection round at the end of a breeding cycle, the parents of these hybrid combinations, the number of plants and families in each breeding scheme per generation, the genotypes and genotypic values of the output population, and the population mean of genotypic values. The number of hybrid combinations retained after a breeding cycle primarily depended on the selection method. Simulation results showed that the bulk method retained more hybrid combinations (e.g., F2 = 500, Figure 4). The number of plants and families per generation in each breeding scheme was influenced by both the selection method and the planting scale of the F2 generation. Under the modified pedigree method, the number of plants and families per generation increased with an increase in the F2 planting scale. In contrast, under the bulk method, these numbers remained unchanged despite variations in the F2 planting scale (e.g., single cross, Table S5). This suggests that while the bulk method offers a stable workload and lower economic costs, the pedigree method offers greater controllability over these factors. Under identical environmental conditions, the mean genotypic value of the offspring population selected by the pedigree method was slightly higher than that of the bulk method. However, the bulk method produced a wider dispersion of genotypic values. In all simulations, the mean genotypic values of the progeny populations exceeded those of the initial RIL3613 population (Figure 5), highlighting its strong breeding potential and suitability for developing high-protein soybean varieties (e.g., F2 = 500, Table S6).

### 2.7. Formulation of Breeding Program

The simulation results were significantly influenced by environmental factors, the impact of which on actual breeding cannot be ignored. Therefore, breeding schemes must be tailored to each environment. Target genotypes should be screened based on the genotypic values of the simulated output population. Additionally, the number of target genotypes obtained per simulation and the corresponding hybrid combinations must be recorded (Table S7). The number of target genotypes obtained per simulation ranges from 0 to 1064, while the number of hybrid combinations producing these genotypes ranges from 0 to 419. The choice of breeding scheme affects the acquisition of target genotypes. Thus, in our study, the top three breeding schemes yielding the highest number of target genotypes under each environmental condition were selected as the best for that environment (Table 6). The screening results indicate that all the best schemes employed the pedigree method for selection, with an F2 planting scale predominantly of 800, though some used 500. Different environmental conditions require distinct recurrent parents for optimal breeding outcomes, with varieties possessing a higher number of PSAs being more likely to serve as recurrent parents (for instance, HN2 in E01, HN12 in E05, HN87 in E06, and HN115 in E07). In E03 and E06, the number of SA in the offspring did not increase significantly compared to the initial population. However, the genotypic value did increase. This indicates that these schemes can aggregate SA with larger effect values. These findings suggest that when breeding resources are not a limiting factor, using the pedigree method and maximizing the F2 generation scale can enhance the acquisition of target genotypes.

## 3. Discussion

In this study, the RIL3613 population was used to identify QTLs and QEIs associated with soybean SPC. The BIP model identified 19 QTLs across 22 environments. Four QTLs had a phenotypic contribution exceeding 10%, classifying them as the major-effect QTL. Using the SoyBase database, these 19 QTLs were compared with 241 previously mapped seed protein-related QTLs. Eight QTLs overlapped with or were included in prior findings [36,37,38,39,40,41,42], while the remaining 11 were identified as novel, validating the reliability of the QEI mapping results. Through parental sequence comparisons and haplotype analysis, a key gene, *Glyma.12G231400*, associated with soybean protein content, was predicted within the 38,995,090–39,293,825 bp region on chromosome 12. This gene is annotated as BEH4 (BES1/BZR1 homolog 4), a homolog of the BHLH transcription factors BRASSINOSTEROID INSENSITIVE 1 (BES1) and BRASSINAZOLE RESISTANT 1 (BZR1), which are critical in brassinosteroid (BR) signaling. BRs are common plant hormones, and previous studies indicated that BEH1 and BEH2 are regulated by brassinolide (BL) in Arabidopsis [43]. BL, the most prevalent BR, has been shown to increase SPC in common beans [44,45]. The overexpression of BEH4 in tomatoes enhances the expression of genes involved in nitrogen uptake and assimilation [44]. Therefore, BEH4 is likely to promote soybean SPC synthesis and accumulation by modulating BL and nitrogen absorption.

Breeders are increasingly leveraging the expanding wealth of published gene and QTL data, along with the widespread adoption of marker-assisted selection, to accelerate crop improvement. While most QTL mapping efforts have focused on single-environment QTL detection, multi-environment trial (MET) QTL mapping and the detection and modeling of QEIs have received less attention [46]. QEIs can be studied when genetic populations are grown across multiple locations or years, providing invaluable insights for both breeders and geneticists. Based on QTL mapping results, breeders can design optimal genotypes with favorable alleles and implement marker-assisted selection more effectively. Stable QTLs for agronomic traits are applicable across diverse environments, whereas environment-specific QTLs are useful for targeted environments [47].

In this study, 97 QEIs were identified using the ICIM method within the MET module. These QEIs were compared with 241 previously mapped seed protein-related QTLs in the SoyBase database. Forty QEIs overlapped with or were included in prior research findings [36,37,39,40,42,48,49,50,51,52,53,54,55,56,57,58], validating the reliability of the QEI mapping results (Figure S2).

Additionally, ISB, an application module within Blib, was used to simulate pure-line variety development in plants. Key elements for these simulations included environmental and breeding target trait genetic models, parental populations, and breeding methods [59]. Genetic models were primarily constructed based on previous genetic studies. The QEIs identified in this study are minor in effect, stable, and widely distributed across the soybean genome, with reliable localization results, making them suitable for genetic model construction. The SPC of the RIL3613 population used in this study exhibited a normal or near-normal distribution across all 11 simulated environments, with transgressive segregation observed within the population, indicating its suitability for breeding based on soybean SPC traits. To maximize the breeding potential of the RIL3613 population, single cross, backcross, pedigree, and bulk selection methods were applied simultaneously, and a half-diallel cross design was used to simulate all possible cross combinations within the population. Consequently, the breeding simulation results and the proposed optimal breeding strategies in this study are considered reliable.

Environmental factors can significantly influence breeding outcomes, making genotype selection for local conditions essential to enhancing soybean protein content [60,61]. Therefore, a key objective for breeders is to develop genotypes suited to a specific set of environments, termed the “Target Population of Environments” (TPE), which includes a defined range of farms and expected growing seasons [62]. This study designed optimal breeding schemes for individual environments within the TPE, ensuring that each scheme produced target genotypes suited to its respective conditions. However, the study characterized environments solely based on heritability. To improve breeding strategies and variety recommendations, future research should focus on precisely describing climatic stress patterns that may influence environments [63]. Analyzing 25 years of data across 35 regions, Beillouin et al. used historical yield records and weather databases to identify four climatic factor combinations affecting barley crops in the French barley belt, with important implications for local genotype adaptation strategies [64]. Similarly, Heinemann et al. integrated a generalized additive model (GAM), environmental covariates (ECs), and grain yield (GY) data from 18 years of historical breeding trials to develop an “environmental forecasting” approach. This approach predicts the optimal EC thresholds for each production scenario (four regions, three seasons, and two grain types) and their respective contributions to GY adaptation, revealing strong interactions between developmental stages, seasons, and regions due to the nonlinear effects of air temperature, solar radiation, and rainfall [65]. Similarly, precise environmental characterization can similarly enhance breeding simulations, leading to more reliable breeding schemes based on simulation results.

The breeding strategy developed in this study could be applied to various environments. This would involve evaluating parental populations in the target environment, performing QTL mapping, and using QTL and population data for breeding simulations. The simulation results could then guide the design of breeding strategies. This study offers new insights for designing soybean breeding programs.

## 4. Materials and Methods

### 4.1. Plant Populations

Two soybean varieties with large differences in seed protein content, i.e., DongnongL13 (45.50%), derived from a cross between Heinong40 and Jiujiao5640, and Heihe36 (39.80%), derived from a cross between Bei 89-7 and Jiusan90-66, were used as parents for crossbreeding in 2008 in Harbin, Heilongjiang Province (E 126.63°, N 45.75°). The F1 generation was planted in Acheng City, Hainan Province (E 109.00°, N 17.50°) in the winter of the same year. After five consecutive generations (2010–2014) of alternate planting in NEAU and Acheng, a population of 120 recombinant inbred lines (RILs) was obtained and used for linkage analysis [66].

### 4.2. Field Trials and Phenotypic Measurement

In this study, the RIL3613 population was grown under 22 different environmental conditions. Field experiments followed a randomized block design with three replications. Each plot consisted of a single row, measuring 5 m in length and 0.67 m in width. Details of sowing dates, planting density, and fertilization rates are provided in Table 7. All other field management practices adhered to local soybean production standards. At maturity, 10 uniformly grown plants from the middle of each row were manually harvested. Once the threshed soybean seeds reached ~13% moisture content (seed dry weight), soybean SPC was measured using a near-infrared grain analyzer (FOSS Infratec 1241, Denmark). The average of three replicates per sample was used as phenotypic data for subsequent analyses (Table 7).

### 4.3. Statistical Analysis of Phenotype Data

Frequency distribution histograms were generated using the average protein content phenotypic values from three replicates per environment, followed by descriptive statistics being used to calculate the mean, standard deviation, minimum, maximum, skewness, kurtosis, and coefficient of variation. An analysis of variance was conducted on repeated phenotypic values of three proteins across multiple environments, and broad-sense heritability was estimated. The statistical model for multi-environment variance analysis is as follows:

*xij* = *μ* + *Gi* + *Ej* + *GEij* + *εij*

where *μ* represents the overall population mean, *G_i_* signifies the effect of the *i*th genotype, *Ej* denotes the effect of the *j*th environment, *GEij* represents the genotype × environment interaction effect, and *εij* is the error effect, following a distribution of *N*(*0*, *σ*^2^).

The broad-sense heritability in multiple environments was calculated via the following formula:

$$h^{2}=\frac{\sigma_{G}^{2}}{\sigma_{G}^{2}+\frac{\sigma_{\mathrm{GE}}^{2}}{e}+\frac{\sigma^{2}}{\mathrm{er}}}$$

where *h*^2^ is the broad-sense heritability of genotype × environment interaction, $\sigma_{G}^{2}$ is the genotypic variance, $\sigma_{\mathrm{GE}}^{2}$ is the variance due to genotype × environment interaction, *σ*^2^ is the error variance, *e* is the number of environments, *r* is the number of replicates within each environment. The data were analyzed using the Proc Mixed procedure in SAS software (SAS 9.4M9 Institute, Cary, NC, USA).

### 4.4. SNP Genotyping and Genetic Map Construction

In a previous study, all RIL3613 lines were genotyped using the SoySNP660K BeadChip, yielding 108,342 SNPs makers across 20 chromosomes. A bin-based linkage map was then constructed using QTL IciMapping v4.2. The total map length was 2969.84 cM, with an average marker spacing of 1.33 cM. The average length of the RIL3613 linkage map was 148.50 cM, with an average of 111.25 markers [34].

### 4.5. QTL Localization

This study employed the BIP and MET models from QTL IciMapping v4.2 for a multi-environment joint analysis. Inclusive composite interval mapping (ICIM-ADD) was used to detect additive effects of SPC. The scan step was set to 1.00 cM, the logarithm of odds (LOD) threshold to 2.50, and the *p*-value for entering variables (PIN) to 0.001. QTLs were named following the method of McCouch [67]. QTLs identified by the BIP model were used to predict candidate genes, while QEIs identified by the MET model were used for breeding simulation.

### 4.6. Candidate Gene Prediction

For the QTLs identified by the BIP model, candidate genes were screened within QTL intervals where phenotypic contributions exceeded 10%, using the Phytozome database. A sequence comparison of all genes between parental lines was conducted based on the resequencing data of the RIL3613 population, retaining genes with nonsynonymous mutations. Subsequently, genes potentially associated with soybean SPC were then selected for haplotype analysis to determine the final candidate genes.

### 4.7. Haplotype Analysis of Candidate Genes

Based on the CDS missense mutation sites of the candidate gene, two haplotypes, i.e., HapI and HapII, were identified, and primers were designed. Leaf DNA was extracted from homozygous lines within the RIL3613 population, and the target fragment was cloned and sequenced. Subsequently, correlation analysis was then performed using phenotypic and sequencing data from the test samples. Furthermore, haplotype analysis was conducted on the candidate gene with superior haplotypes using genotypic data from 2898 soybean germplasm resources in the SoyOmics database and the corresponding phenotypic data, thereby validating haplotype function. Haplotype typing of the germplasm resources was performed using VCFtools v1.16 in the Linux system terminal.

### 4.8. Breeding Simulations Based on the Blib Platform

**Figure 6:**
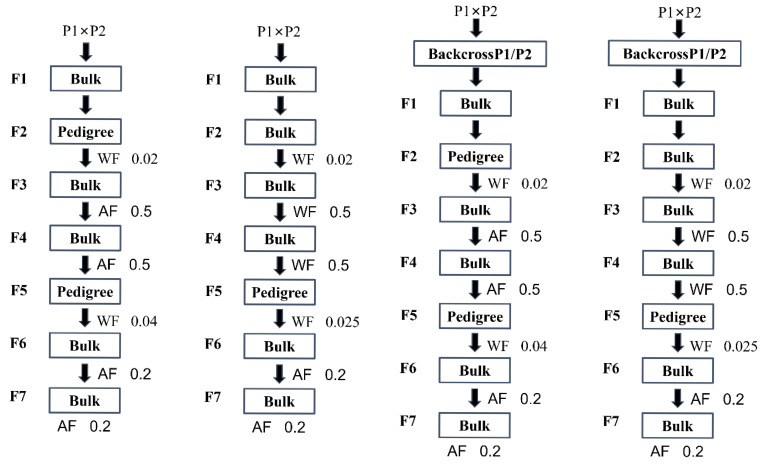
Flowchart of six breeding schemes. The six breeding schemes, from left to right, are PedSC, BlkSC, PedBC1P1/PedBC1P2, and BlkBC1P1/BlkBC1P2. “pedigree” represents after within-family selection; each retained individual in one family was propagated and harvested to make one pedigree family in the next generation of breeding. “bulk” represents after within-family selection; all retained individuals in one family were propagated and harvested together to make one bulk family in the next generation of breeding. In the F1 generation, ten plants were grown for each hybrid variety. In the F2 generation, each line was planted with 200, 500, or 800 individuals. For the F3 and F4 generations, thirty plants were grown per line, while in the F5 to F7 generations, fifty plants were grown for each line. AF denotes selection among families, WF denotes selection within families, with the fractional values representing the selection ratios. In each generation, the highest phenotypic families are selected according to the specified ratios.

A half-diallel cross was designed, where 120 parental lines were paired, yielding 7140 hybrid combinations. Hybridization methods included single crosses and backcrosses, while selection methods involved either pedigree or bulk selection. Six breeding schemes were developed based on different hybridization and selection methods: PedSC, BlkSC, PedBC1P1, PedBC1P2, BlkBC1P1, and BlkBC1P2 (Figure 6). The initial parental population and genetic model were constructed using QEI information and the genotypes of various RIL lines. The ISB’s B4L functionality was employed to simulate these breeding schemes. A virtual QEI, whose genotype value equalled the total effect of all remaining quantitative trait loci associated with protein content, was incorporated into the simulation to enhance accuracy in predicting soybean protein levels. The number of F2 generation planting lines significantly influenced breeding outcomes; therefore, the F2 planting scale was set at 200, 500, or 800 plants per line. Simulations for all six breeding schemes were conducted across multiple environments to assess differences among progeny populations under varying conditions. The design of breeding schemes required specifying a target genotype. Given the large number of identified QEIs, the breeding target was adjusted to a target genotypic value. The breeding objective was defined as the highest genotypic value within the initial population across different environments. Virtual progeny with genotypic values meeting or exceeding this target were considered the target genotype.

## Figures and Tables

**Table 1:** Description and analysis of protein content of RIL3613 population.

	Parent		RIL3613 Population						
	P_1_ ^a^	P_2_	Mean	STD	Minimum (%)	Maximum (%)	Skew	Kurt	CV ^b^ (%)
E01	42.11	39.77	43.48	2.05	38.00	47.37	−0.39	−0.37	4.70
E02	42.63	39.06	42.30	2.26	36.74	46.14	−0.27	−0.69	5.34
E03	43.59	40.02	42.64	1.28	38.60	44.80	−0.53	−0.28	3.01
E04	43.15	38.56	41.74	1.45	37.59	44.77	−0.21	−0.24	3.48
E05	43.06	38.16	41.82	1.38	37.50	45.00	−0.54	0.88	3.31
E06	42.72	38.65	41.44	1.21	38.20	44.20	−0.20	−0.26	2.91
E07	42.77	39.55	41.54	1.48	38.00	45.30	−0.19	−0.19	3.57
E08	42.03	38.97	41.53	1.46	37.50	44.60	−0.23	−0.05	3.52
E09	42.95	37.64	41.31	1.31	36.20	44.00	−1.12	2.71	3.18
E10	43.36	39.12	42.06	1.28	37.80	45.00	−0.84	1.11	3.03
E11	43.10	39.99	42.22	1.12	38.80	44.60	−0.35	−0.11	2.66
E12	43.30	39.23	42.22	1.49	37.40	44.70	−0.82	0.69	3.52
E13	41.99	38.25	41.44	1.39	36.60	44.20	−0.65	0.50	3.37
E14	42.32	39.48	41.15	1.35	36.90	44.50	−0.44	0.80	3.28
E15	42.03	37.99	41.62	1.19	37.40	44.00	−0.79	0.84	2.86
E16	42.22	37.56	41.21	1.46	36.60	46.10	−0.35	0.93	3.54
E17	43.21	39.26	42.54	0.96	38.10	44.30	−1.06	2.62	2.27
E18	43.99	39.15	42.59	0.87	40.50	44.40	−0.32	−0.26	2.04
E19	44.00	39.68	42.41	0.93	38.90	44.90	−0.46	1.39	2.18
E20	43.26	38.12	42.09	0.99	38.60	44.20	−0.50	1.02	2.34
E21	42.96	38.62	41.91	1.36	36.50	44.30	−1.10	2.05	3.24
E22	42.58	39.25	41.92	1.32	37.80	45.20	−0.19	0.51	3.16

Specific environmental data for E01 to E22 are presented in Table 7. ^a^ Parents: P_1_, female cultivar “Dongnong L13”; P_2_, male cultivar “Heihe 36”. ^b^ Coefficient of variation.

**Table 2:** Analysis of variance protein content across 22 environments.

Source	DF	SS	MS	F	Pr (>F)	Significance	Variance
Environment (E)	21	2340	111.45	77.777	<2 × 10^−16^	***	
Genotype(G)	119	2989	25.12	17.531	<2 × 10^−16^	***	0.331
Block	39	59	1.50	1.050	0.386		
G × E	2345	10,998	4.69	3.273	<2 × 10^−16^	***	1.086
Residuals	4933	7069	1.43				1.433
h^2^							0.823

Signif. codes: <0.001 ‘***’. h^2^ represents heritability.

**Table 3:** Four QTL detected in multiple environments.

QTL Name	Env.	Chr.	Marker Interval	LOD ^a^	PVE (%) ^b^	ADD ^c^	Physical Region (bp)
qPR-1-1	E21	1	36c01042~36c01043	3.0802	12.3745	−0.4787	38,760,401~41,326,475
qPR-1-3	E20	1	36c01048~36c01049	3.3665	12.2999	−0.3506	42,339,555~43,008,300
qPR-12-1	E08	12	36c12076~36c12077	2.8396	12.2795	0.5115	38,995,090~39,293,825
qPR-14-1	E19	14	36c14059~36c14058	4.8113	15.8298	0.4002	9,898,619~10,293,987

^a^ LOD, logarithm of odds. ^b^ PVE, phenotypic variation explained by QTL. ^c^ ADD, additive effect.

**Table 4:** Detailed information on four candidate genes related to protein content.

QTL Name	Gene Name	Chr.	Position	Annotation
qPR-1-1	*Glyma.01G114100*	1	39,440,337–39,440,694	rubisco activase
qPR-1-3	*Glyma.01G123300*	1	42,526,283–42,535,486	BCL-2-associated athanogene 4
qPR-12-1	*Glyma.12G231400*	12	39,138,373–39,142,508	BES1/BZR1 homolog 4

**Table 5:** SNP variation in the CDS region of candidate genes.

Gene	Parents	Position/bp (Chromosome 1)	
		39440429	39440646
*Glyma.01G114100*	Donong L13	T(Lys)	C(Met)
	Heihe36	A(Met)	A(Ile)
		**Position/bp (Chromosome 1)**	
		42529392	
*Glyma.01G123300*	Donong L13	G(Glt)	
	Heihe36	A(Lys)	
		**Position/bp (Chromosome 12)**	
		39141373	
*Glyma.12G231400*	Donong L13	A(His)	
	Heihe36	C(Pro)	

**Table 6:** Optimal breeding strategies for each environment.

Environment	Selection Method	F2 Generation Planting Scale	Cross Combination	Number of Target Genotype	Number of Superior Alleles	Interval
E01	Ped	800	(HN2 × HN118) × HN2	24	52~77	42.8318~45.954
			HN2 × HN120	23	68~74	44.093~45.3332
			(HN4 × HN120) × HN120	19	67~74	43.882~45.5186
E02	Ped	800	(HN14 × HN114) × HN14	10	59~67	41.4378~42.8189
			HN14 × HN117	9	61~66	41.6547~42.5099
			(HN12 × HN117) × HN117	9	59~67	41.8867~42.8811
			(HN14 × HN117) × HN117	9	59~64	41.6028~42.1985
E03	Ped	800	HN34 × HN116	6	61~67	42.4901~43.5157
			HN12 × HN116	6	54~65	42.0977~43.5478
			HN44 × HN105	6	61~67	42.4901~43.5157
			(HN32 × HN116) × HN32	6	58~66	42.6208~43.9469
E04	Ped	800	(HN38 × HN119) × HN38	8	66~70	46.4045~48.0717
		500	HN3 × HN12	7	67~74	46.1507~46.8833
		800	HN3 × HN12	6	66~73	45.9351~47.1123
E05	Ped	800	HN1 × HN12	12	75~78	43.1178~43.4624
		800	(HN1 × HN12) × HN12	12	74~78	42.991~43.4624
		500	(HN1 × HN12) × HN12	11	76~78	43.2866~43.4624
E06	Ped	800	(HN87 × HN118) × HN87	21	54~63	38.4959~42.3515
			(HN4 × HN118) × HN118	16	41~57	37.7403~41.5515
			(HN3 × HN118) × HN118	16	46~57	38.3227~41.3913
E07	Ped	800	(HN61 × HN115) × HN115	7	74~78	44.0076~44.71
			(HN48 × HN115) × HN115	4	70~74	42.8634~44.343
			(HN115 × HN118) × HN115	4	62~75	41.9844~45.4756
E08	Ped	800	(HN27 × HN118) × HN27	6	60~67	42.9358~44.1906
			(HN74 × HN104) × HN74	5	63~71	42.3392~44.3778
			(HN11 × HN21) × HN11	5	65~72	42.8532~43.6847
			(HN11 × HN108) × HN11	5	62~69	42.0788~43.6776
			(HN11 × HN107) × HN11	5	68~69	43.0674~43.3582
			(HN16 × HN80) × HN16	5	63~68	42.4404~43.2367
E09	Ped	800	(HN115 × HN118) × HN115	23	64~73	42.3694~44.471
			(HN115 × HN120) × HN115	16	68~75	42.1194~44.5158
			HN115 × HN118	10	57~73	40.7384~44.6431
E10	Ped	800	(HN67 × HN118) × HN118	12	64~69	41.3527~43.0921
			HN107 × HN118	10	62~70	40.7067~43.8515
			(HN107 × HN114) × HN107	9	56~64	41.2065~43.0447
			(HN80 × HN118) × HN80	9	54~68	39.8393~42.6609
			(HN103 × HN115) × HN103	9	62~69	41.4689~42.0991
E11	Ped	800	(HN2 × HN118) × HN2	17	72~80	45.6911~48.2653
			(HN2 × HN120) × HN2	14	72~79	46.0565~47.7315
			(HN2 × HN116) × HN2	11	74~78	45.9251~47.0337

**Table 7:** Field management methods in 22 environments.

Environment	Year	Location	Sowing Date	Planting Density (×104 plant/hm^2^)	(N/P_2_O_5_/K_2_O) (kg/hm^2^)
E01	2013	Keshan	13-May	30	75/150/75
E02	2014	Harbin	10-May	22	75/150/75
E03	2015	Harbin	10-May	30	75/150/75
E04	2015	Keshan	12-May	35	75/150/75
E05	2016	Acheng	25-May	22	75/150/75
E06	2016	Acheng	10-May	22	75/150/75
E07	2016	Acheng	10-May	30	75/150/75
E08	2016	Acheng	10-May	22	0/150/75
E09	2016	Shuangcheng	28-May	22	75/150/75
E10	2016	Shuangcheng	12-May	22	75/150/75
E11	2016	Shuangcheng	12-May	30	75/150/75
E12	2016	Shuangcheng	12-May	22	0/150/75
E13	2016	Harbin	23-May	22	75/150/75
E14	2016	Harbin	10-May	22	75/150/75
E15	2016	Harbin	10-May	30	75/150/75
E16	2016	Harbin	10-May	22	0/150/75
E17	2017	Shuangcheng	8-May	22	75/150/75
E18	2018	Acheng	9-May	22	75/150/75
E19	2019	Harbin	10-May	22	75/150/75
E20	2019	Shuangyashan	13-May	25	75/150/75
E21	2020	Harbin	10-May	22	75/150/75
E22	2020	Shuangyashan	14-May	25	75/150/75

## Data Availability

The original contributions presented in the study are included in the article/Supplementary Materials, further inquiries can be directed to the corresponding authors.

## Supplementary Materials

The following supporting information can be downloaded at: https://www.mdpi.com/article/10.3390/plants14142117/s1, Figure S1: Frequency histogram of protein content of RIL 3613 in 22 environments; Figure S2: Distribution of QTL of RIL3613 protein content on genome map. The blue font indicates the protein content QTL located in previous studies. Table S1: Details of 19 QTLS for RIL 3613 linkage analysis; Table S2: Haplotype sequencing primers; Table S3: Details of 97 QTLS for RIL 3613 linkage analysis; Table S4: The number of advantageous alleles and genotype values across 120 lines in 11 environments; Table S5: The number of families and plants selected before and after each breeding generation; Table S6: The population means, genotype value variance, range and broad-sense heritability of the progeny population and the initial population. Table S7: Information on target genotypes obtained from various breeding schemes.

## Abbreviations

The following abbreviations are used in this manuscript:

BIP	Bi-parental populations
MET	Multi-environment trials
QTL	Quantitative trait locus
QEI	QTL × environment interactions
GWAS	Genome-wide association studies
LOD	Logarithm of odd
CDS	Coding sequence
ICIM	Inclusive composite interval mapping
