# Supplementary material for: Genetic Basis and Simulated Breeding Strategies for Enhancing Soybean Seed Protein Content Across Multiple Environments
Source: Plants (Basel). 2025 Jul 9;14(14):2117. doi: 10.3390/plants14142117 (PMC12298690; doi:10.3390/plants14142117)
Supplement: Supplementary file 1 [file plants-14-02117-s001.zip › Supplementary Figures.pdf]

Supplementary

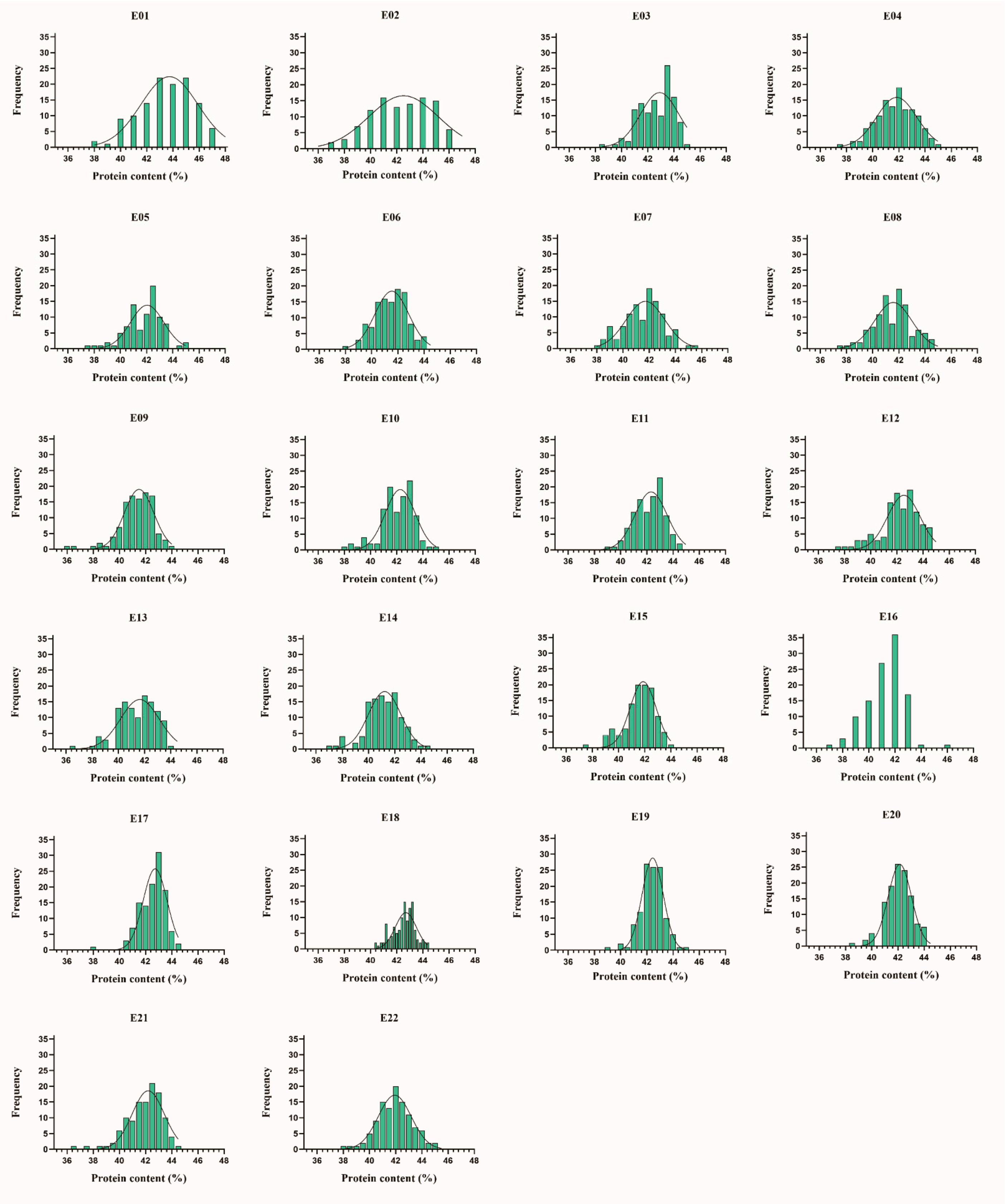

Figure S1. Frequency histogram of protein content of RIL 3613 in 22 environments.

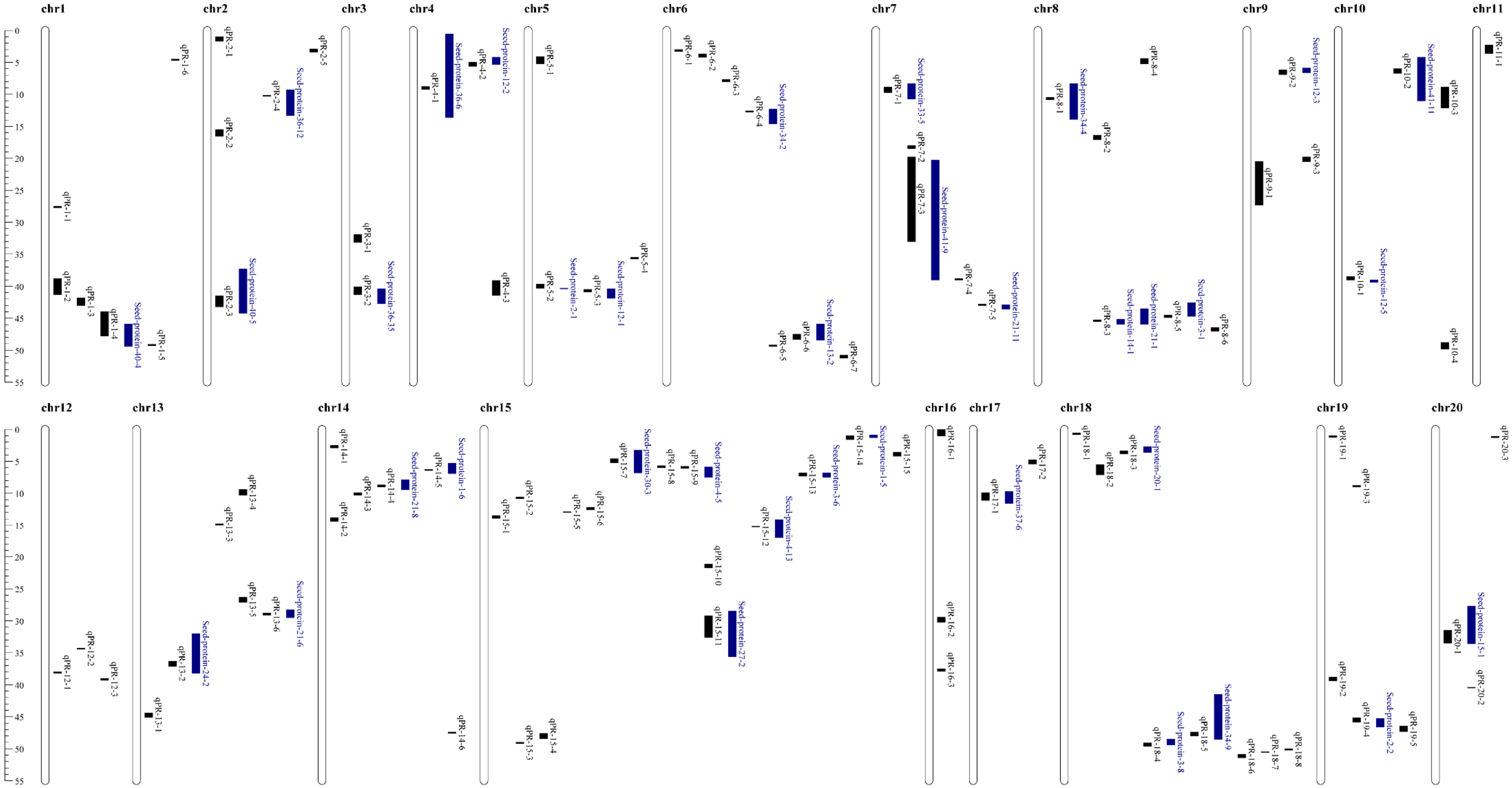

Figure S2**.** Distribution of QTL of RIL3613 protein content on genome map. The blue font indicates the protein content QTL located in previous studies.
